# Efficacy and safety of recombinant human endostatin combined with radiotherapy or chemoradiotherapy in patients with locally advanced non-small cell lung cancer: a pooled analysis

**DOI:** 10.1186/s13014-020-01646-9

**Published:** 2020-08-24

**Authors:** Shu-Ling Zhang, Cheng-Bo Han, Li Sun, Le-Tian Huang, Jie-Tao Ma

**Affiliations:** Department of Oncology, Shengjing Hospital of China Medical University, Shenyang, 110004 China

**Keywords:** Chemoradiotherapy, Endostatin, Non-small cell lung cancer, Radiotherapy

## Abstract

**Purpose:**

To assess the efficacy and safety of recombinant human endostatin in combination with radiotherapy (RT) or concurrent chemoradiotherapy (CCRT) in patients with locally advanced non-small cell lung cancer (LA-NSCLC).

**Methods:**

We searched eligible literature in available databases using combinations of the following search terms: lung cancer, endostatin or endostar, radiotherapy or radiation therapy or chemoradiotherapy. The inclusion criteria were: prospective or retrospective (including single-arm) studies that evaluated the efficacy and safety of endostatin plus radiotherapy (ERT) or concurrent chemoradiotherapy (ECRT) in patients with LA-NSCLC. Primary outcomes included the following: objective response rate (ORR), local control rates (LCR), overall survival (OS), progression-free survival (PFS), and adverse events (AEs). Tests of heterogeneity, sensitivity, and publication bias were performed.

**Results:**

A total of 271 patients with LA-NSCLC from 7 studies were enrolled, including six prospective trials and one retrospective study. The pooled median PFS was 11.3 months overall, 11.2 months in the ECRT group, and 11.8 months in the ERT group. Pooled median OS and ORR were 18.9 months and 77.2% overall, 18.4 months and 77.5% in the ECRT group, and 19.6 months and 76.1% in the ERT group, respectively. The incidences of major grade ≥ 3 AEs for all patients, subgroups of ECRT and ERT were 10.9% vs 11.9% vs 9.4% for radiation pneumonitis, 11.6% vs 12.2% vs 9.4% for radiation esophagitis, 35.5% vs 43.4% vs 0 for leukopenia, 27.8% vs 40.7% vs 2.1% for neutropenia, and 10.5% vs 12.3% vs 2.1% for anemia.

**Conclusions:**

Combined endostatin with RT or CCRT is effective and well tolerated in treating LA-NSCLC, and less toxicities occur. Further validation through prospective randomized control trials is required.

## Introduction

Lung cancer is the most common cancer type worldwide [1], and non-small cell lung cancer (NSCLC) is the most common form (80–85%) [2]. At the time of initial diagnosis, approximately one-third of patients with NSCLC present with locally advanced NSCLC (LA-NSCLC) [3]. Furthermore, about 70% of LA-NSCLCs are unresectable, and chemoradiotherapy (CRT) was the recommended standard care for these patients [4, 5]. No significant progress in the treatment of LA-NSCLC was made for many years until the PACIFIC study confirmed that consolidation therapy with durvalumab (a monoclonal antibody that blocks interactions of programmed cell death ligand 1 with the PD-1 receptor) further improved survival following CRT [6–8].

Previous studies indicated that a hypoxic tumor microenvironment contributes not only to resistance of tumor cells to chemoradiation but also promotes metastasis [9, 10], and tumor oxygenation is essential for effective application of radiotherapy (RT) or CRT [11]. Therefore, novel treatments that enhance radiosensitivity by improving the hypoxic microenvironment are urgently needed. Prior to the findings of the PACIFIC study, researchers explored whether patients with LA-NSCLC could benefit from anti-angiogenic drugs combined with RT or CRT. However, earlier studies showed that administration of bevacizumab along with thoracic RT led to a high incidence of pulmonary toxicity, including radiation pneumonitis, hemoptysis and tracheoesophageal fistulae, in patients with stage III NSCLC [12, 13]. Therefore, concurrent bevacizumab with thoracic RT is unlikely to be further pursued as a treatment option for stage III NSCLC.

Preclinical studies have demonstrated that endostatin (a broad-spectrum angiogenesis inhibitor) is able to normalize tumor vasculature, alleviate hypoxia and increase tumor sensitivity to radiation [14, 15]. Several studies have indicated enhanced efficacy and tolerable toxicity of endostatin combined with thoracic RT or CRT for patients with LA-NSCLC [16–18]. However, the reported studies to date are mostly retrospective or single arm studies with limited patient enrolment. In the present study, we performed a pooled analysis to assess the clinical efficacy and safety of endostatin combined with RT or concurrent chemoradiotherapy (CCRT) in patients with LA-NSCLC.

## Materials and methods

### Search strategy

We conducted a systematic search for available articles, both in published and abstract forms of PubMed, OVID, Web of SCI, EMBASE, Google Scholar, Cochrane Library, Chinese National Knowledge Infrastructure, and Wanfang databases. The final literature search was performed on June 30, 2019, using the following search terms: “lung cancer” AND (endostatin OR endostar) AND (radiotherapy OR radiation therapy OR chemoradiotherapy). Manual updates of abstracts presented till the 2019 meetings, such as American Society of Clinical Oncology, European Society for Medical Oncology, World Conference of Lung Cancer, and American Society for Therapeutic Radiology and Oncology were additionally performed.

### Study selection and search strategy

Studies that met the following inclusion criteria were included in the pooled analysis: 1) prospective or retrospective (including single-arm) studies that evaluated the efficacy and safety of endostatin plus radiotherapy (ERT) or concurrent chemoradiotherapy (ECRT) in patients with LA-NSCLC; 2) studies with primary outcomes reporting at least one of the following endpoints: objective response rate (ORR), progression-free survival (PFS) and overall survival (OS), and local control rates (LCR), or adverse events (AEs) based on Common Terminology Criteria for Adverse Events version 3.0 or 4.0; 3) number of cases included for study was ≥10; 4) articles or abstracts were written in English. After the selection process, the remaining titles and abstracts were screened for relevance independently by two authors. Full-text articles and meeting abstracts were finally reviewed for all studies that met the inclusion criteria.

### Data extraction and quality assessment

Data were extracted independently by two reviewers according to the inclusion criteria. Discrepancies were resolved by discussing with a third reviewer. Each reviewer extracted data including author name, the publication years of the studies, number of patients, patient characteristics, treatment regimen, radiotherapy dosage, the method of endostatin administration, ORR, PFS, OS, LCR and AEs. The Jadad scale [19] and Newcastle Ottawa Scale [20] were used to assess the quality of the included studies.

### Statistical analysis

Statistical analyses were conducted using Comprehensive Meta-Analysis (version 3.0) software (Biostat Inc., NJ, USA). For dichotomous variables, such as OS rates, PFS rates, ORR, LCR and AEs, we calculated the raw proportion of events divided by the total number of clinically evaluable patients. Additionally, we calculated weighted pooled rates of events by the number of clinically evaluable patients using a random effects model to account for heterogeneity in study size and the large variations in proportion. Median pooled weighted OS and PFS were calculated with descriptive statistics. Subgroup analysis was performed per type of treatment regimen (ERT or ECRT).

### Publication bias and sensitivity analysis

The potential for publication bias in reported ORR values was assessed by funnel plots, with the appropriate accuracy intervals. Sensitivity analyses were performed for the results for ORR based on the leave-one-out approach.

## Results

### Literature search

**Fig. 1:**
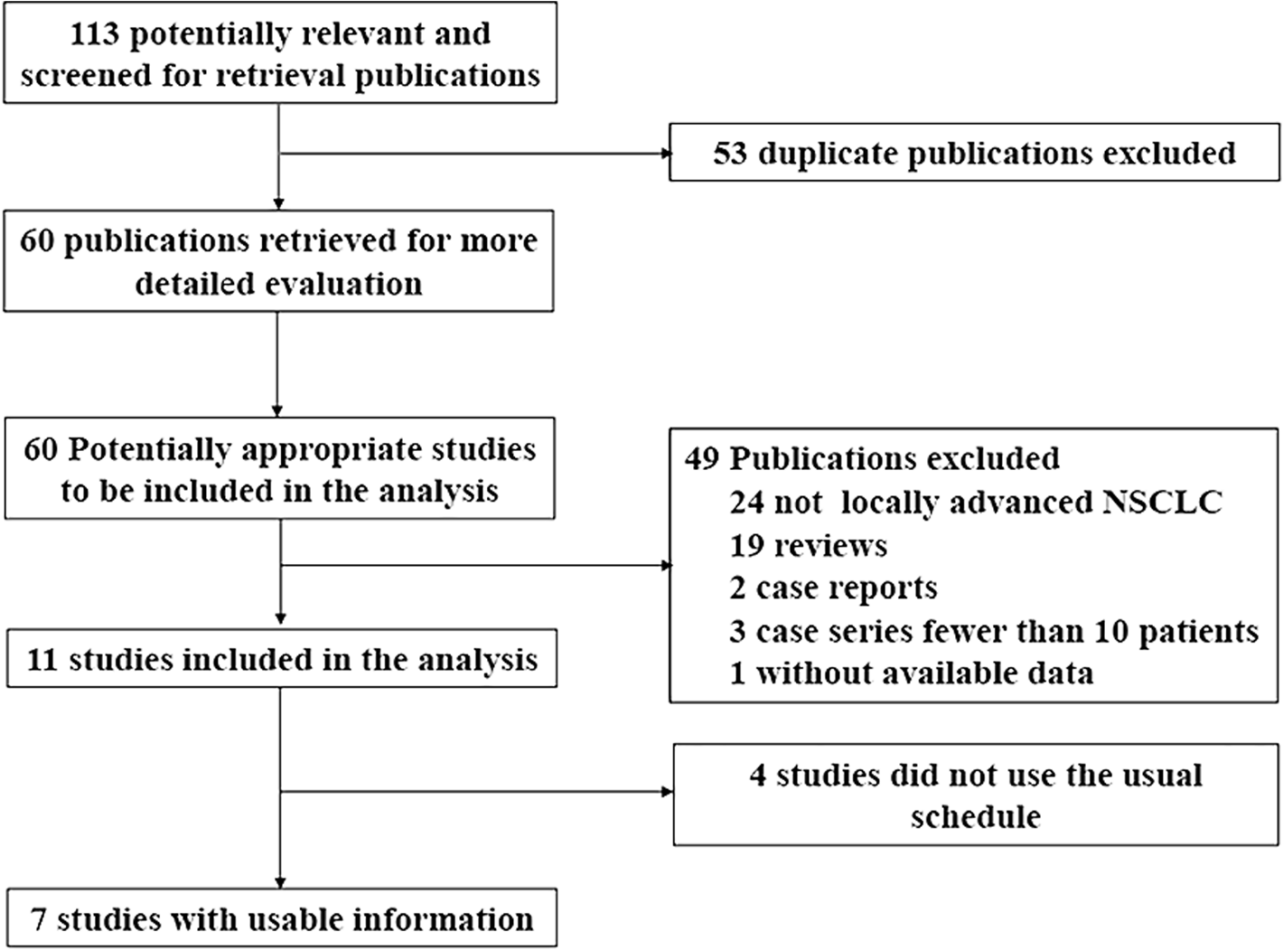
Overview of study search and selection

Figure 1 depicts a flowchart of the literature search procedure. Overall, 113 records were identified using the search strategy and 102 records excluded after screening the titles and abstracts. Among the remaining 11 potentially relevant studies, four were excluded due to endostatin administration via arterial infusion or discontinuation of endostatin in the first cycle during RT. Finally, seven studies [16, 18, 21–25] involving 271 patients were pooled for analysis.

### Included studies and patient characteristics

The characteristics of the selected studies are summarized in Table 1. The included studies comprised three prospective cohort studies, three single-arm prospective studies and one single-arm retrospective study. Follow-up data were available for five studies, with a median follow-up period between 20.0 and 37.1 months. In total, 212 evaluable patients in four studies received endostatin combined with CCRT (ECRT) and 59 evaluable patients in three studies received endostatin combined with single RT (ERT). Patients received a total dose of 60–68 Gy in 30–34 fractions for 6–7 weeks. However, the methods of endostatin treatment differed among studies, including continuous intravenous pumping (CIV) of endostatin (7.5 mg/m^2^/day) over 5 days, administration of endostatin (7.5 mg/m^2^/day) over 4 h for 7 days at weeks 1, 3, 5, and 7 or via an endostatin intravenous drip (IV) (15 mg/day) for 14 days per 3 weeks, etc. Almost all included patients had unresectable LA-NSCLC at the time of study entry. The median patient age ranged from 56 to 76 years.

**Table 1:** Characteristics of the included studies

Study	Published year	Study type	No. of cases	Endpoints	Treatment regimen	Radiation dose (Gy)	Endostatin usage	Total duration of endostatin
Jiang [16]	2012	Prospective cohort study	25	1-, 2-yr OS rate, 1-, 2-yr LCR, OS, ORR, AEs	ERT	60	15 mg/day IV for 7 days during the first week of RT	7 days× 1 cycles
Zhai [18]	2019	Single-arm prospective study	67	1-, 2-, 3-yr PFS/OS rate, PFS,OS, ORR, AEs	ECRT	60–66	7.5 mg/m^2^/day CIV for 5 days before the beginning of RT, and then repeated at week 2, 4, and 6 during RT	5 days× 4 cycles
Sun [21]	2016	Single-arm prospective study	19	ORR, PFS, OS, AEs	ECRT	60–66	7.5 mg/m^2^/day IV for 14 days per 3 weeks during RT	14 days× 2 cycles
Bao [22]	2015	Single-arm prospective study	48	OS, 1-, 2-, 3-yr PFS/OS rate and LCR, PFS, ORR, AEs	ECRT	60–66	7.5 mg/m^2^/day IV for 7 days before the beginning of RT, and then repeated at week 2, 4, and 6 during RT	7 day× 4 cycles
Tang [23]	2016	Single-arm retrospective study	78	PFS, OS, ORR	ECRT	60–66	7.5 mg/m^2^/day IV over 4 h per day for 7 days, or CIV for 5 days, at week 1, 3, 5 and 7, endostatin administrated 1 week prior to CRT	5/7 days× 4 cycles
Wen [24]	2009	Prospective cohort study	14	ORR, PFS, 1-yr OS rate	ERT	66–68	15 mg/day IV during the first three weeks of RT	21 days× 1 cycles
Chen [25]	2017	Prospective cohort study	20	ORR, PFS, OS, AEs	ERT	60–66	15 mg/day IV for 14 days per three weeks during RT	14 day×2 cycles

*OS* Overall survival, *PFS* Progression-free survival, *ORR* Objective response rate, *LCR* Local control rate, *AEs* Adverse events, *ERT* Endostatin combined with radiotherapy, *ECRT* Endostatin combined with concurrent chemoradiotherapy, *yr* Year, *RT* Radiotherapy, *IV* Intravenous injection, *CIV* Continuous intravenous pumping

### Pooled ORR and LCR

**Fig. 2:**
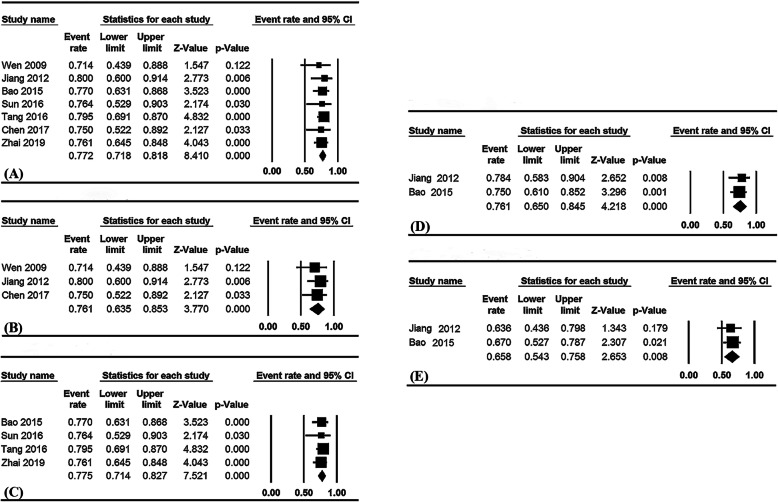
Pooled ORR for all patients (**a**), ERT (**b**) and ECRT (**c**) groups; pooled LCR for all patients, 1-year LCR (**d**) and 2-year LCR (**e**). ORR: objective response rates; ERT: endostatin combined with radiotherapy alone; ECRT: endostatin combined with concurrent chemoradiotherapy; LCR: local control rates

Pooled ORR and LCR data are summarized in Table 2. The pooled overall ORR for the seven studies was 77.2% (95% confidence interval [CI], 71.8–81.8%; I^2^ = 0%, Fig. 2a), 76.1% (95% CI, 63.5–85.3%; I^2^ = 0%, Fig. 2b) in the ERT group and 77.5% (95% CI, 71.4–82.7%; I^2^ = 0%, Fig. 2c) in the ECRT group. Higher ORR was observed in the ERT group, compared with the RT alone group (76.1% vs 61.7%, respectively).

**Table 2:** Pooled efficacy of endostatin combined with radiotherapy or chemoradiotherapy

Endpoints	Group	No. of studies	No. of cases	Weighted pooled data (95%CI)
**Response rate**				
ORR (%)	Overall	7	271	77.2 (71.8–81.8)
	ECRT	4	212	77.5 (71.4–82.7)
	ERT	3	59	76.1 (63.5–85.3)
1-yr LCR (%)	Overall	2	73	76.1 (65.0–84.0)
2-yr LCR (%)	Overall	2	73	65.8 (54.3–75.8)
**Progression-free survival**				
Median PFS (months)	Overall	6	246	11.3
	ECRT	4	212	11.2
	ERT	2	34	11.8
1-yr PFS rate (%)	ECRT	2	115	49.6 (40.5–58.6)
2-yr PFS rate (%)	ECRT	2	115	31.7 (23.8–40.8)
3-yr PFS rate (%)	ECRT	2	115	23.7 (16.7–32.5)
**Overall survival**				
Median OS (months)	Overall	4	142	18.9 (15.3–22.5)
	ECRT	2	97	18.4 (9.7–27.0)
	ERT	2	45	19.6 (16.2–23.1)
1-yr OS rate (%)	Overall	4	154	79.4 (72.1–85.1)
	ECRT	2	115	81.6 (73.5–87.7)
	ERT	2	39	72.8 (55.9–85.0)
2-yr OS rate (%)	Overall	3	140	59.0 (49.7–67.8)
3-yr OS rate (%)	ECRT	2	115	55.7 (45.6–65.6)
	ECRT	2	115	43.9 (29.8–59.0)

*OS* Overall survival, *PFS* Progression-free survival, *ORR* Objective response rate, *LCR* Local control rate, *ERT* Endostatin combined with radiotherapy, *ECRT* Endostatin combined with concurrent chemoradiotherapy, *yr* Year

Only two studies in which the treatment regimens were ECRT and ERT reported LCR data. The pooled 1- and 2-year LCR rates were 76.1% (95% CI, 65.0–84.0%; I^2^ = 0%, Fig. 2d) and 65.8% (95% CI, 54.3–75.8%; I^2^ = 0%, Fig. 2e), respectively.

### Pooled survival

**Fig. 3:**
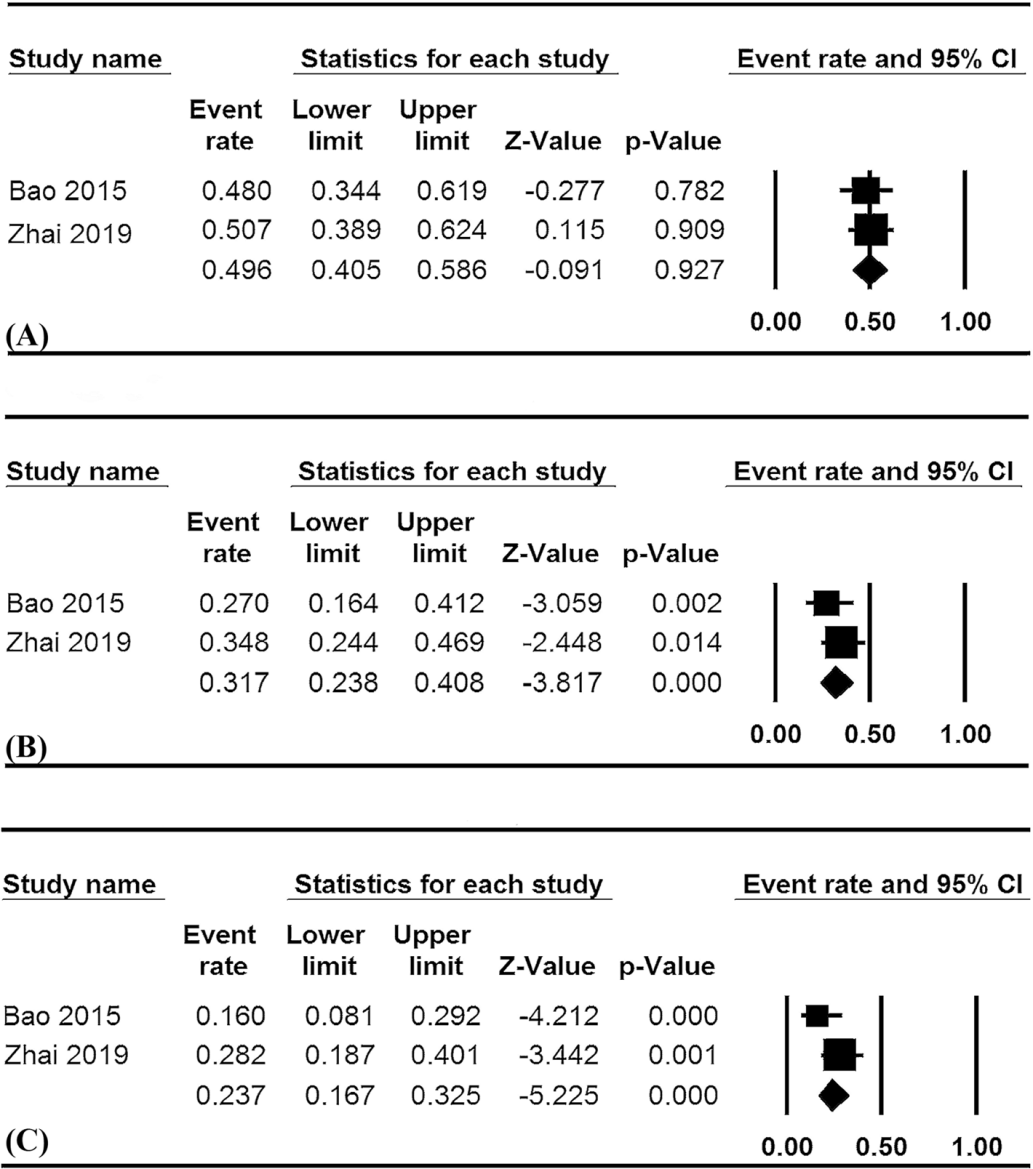
Pooled PFS rates for ECRT group, 1-year (**a**), 2-year (**b**), and 3-year PFS rates (**c**). PFS: progression-free survival; ECRT: endostatin combined with concurrent chemoradiotherapy

The pooled survival data are summarized in Table 2. Only two studies in ECRT group reported PFS rates. The pooled 1-, 2- and 3-year PFS rates were 49.6% (95% CI, 40.5–58.6%; I^2^ = 0%, Fig. 3a), 31.7% (95% CI, 23.8–40.8%; I^2^ = 0%, Fig. 3b), and 23.7% (95% CI, 16.7–32.5%; I^2^ = 56.3%, Fig. 3c), respectively.

**Fig. 4:**
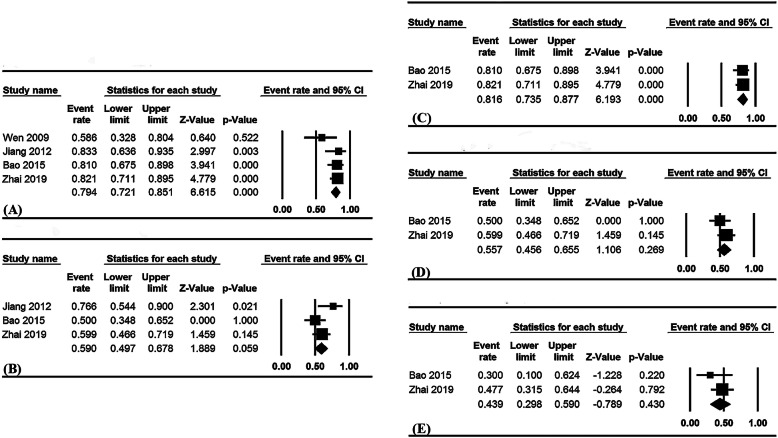
Pooled 1-year (**a**) and 2-year OS rates (**b**) for overall patients; pooled OS rates for the ECRT group, 1-year (**c**), 2-year (**d**), and 3-year (**e**) OS rate. OS: overall survival; ECRT: endostatin combined with concurrent chemoradiotherapy

Four studies documented the 1-year OS rate, three the 2-year OS rate, and two the 3- year OS rate. The overall pooled 1- and 2-year OS rates were 79.4% (95% CI, 72.1–85.1%; I^2^ = 25.2%, Fig. 4a) and 59.0% (95% CI, 49.7–67.8%; I^2^ = 48.1%, Fig. 4b), respectively. Based on stratification by treatment regimens, the pooled 1-, 2- and 3-year OS rates in the ECRT group were 81.6% (95% CI, 73.5–87.7%; I^2^ = 0%, Fig. 4c), 55.7% (95% CI, 45.6–65.6%; I^2^ = 0%, Fig. 4d) and 43.9% (95% CI, 29.8–59.0%; I^2^ = 0%, Fig. 4e); the pooled 1-year OS rate in the ERT group was 72.8% (95% CI, 55.9–85.0%; I^2^ = 63.3%).

Six of the included studies had recorded median PFS values. Patients received ECRT in four of these studies and ERT in the remaining two studies, with only three of the above studies recording both the PFS value and 95% CI. Accordingly, pooled median PFS was calculated by a weighted average of the single study median [26]. The pooled median PFS was recorded as 11.3 months overall, 11.2 months in the ECRT group, and 11.8 months in the ERT group.

OS data and 95% CI were reported in four studies. The overall pooled median OS was 18.9 months (95% CI, 15.3–22.5, I^2^ = 87.6%), 18.4 months (95% CI, 9.7–27.0, I^2^ = 92.6%) in the ECRT group and 19.6 months (95% CI, 16.2–23.1, I^2^ = 78.7%) in the ERT group.

### Safety

The most common AEs documented in the five selected studies, including 179 patients, were radiation pneumonitis, radiation esophagitis, thrombocytopenia, and anemia. Additionally, nausea/vomiting, neutropenia and leukopenia were three commonly observed AEs in three of the above four studies. Pooled data on AEs are summarized in Table 3.

**Table 3:** Pooled adverse events of endostatin combined with radiotherapy or chemoradiotherapy

Events	Grade	Incidence, % (95% CI)		
		Overall	ECRT group	ERT group
Radiation pneumonitis	All	55.9 (31.4–77.9)	50.7 (20.9–80.0)	64.1 (27.3–89.4)
	≥3	10.9 (5.4–20.8)	11.9 (4.5–27.9)	9.4 (3.3–24.0)
Radiation esophagitis	All	77.4 (69.4–83.7)	89.7 (83.1–93.9)	55.5 (40.9–69.3)
	≥3	11.6 (7.6–17.5)	12.2 (7.6–19.0)	9.4 (3.3–24.0)
Neutropenia	All	76.5 (55.6–89.4)	85.7 (78.5–90.7)	25.1 (71.6–89.9)
	≥3	27.8 (14.3–47.0)	40.1 (30.3–50.8)	2.1 (0.3–13.7)
Leukopenia	All	84.5 (49.7–96.8)	91.8 (78.2–97.2)	40
	≥3	35.5 (18.5–57.7)	43.4 (27.2–61.2)	0
Anemia	All	54.7 (34.7–73.3)	70.5 (62.1–77.6)	28.9 (17.6–43.6)
	≥3	10.5 (6.2–17.2)	12.3 (7.6–19.1)	2.1 (0.3–13.7)
Thrombocytopenia	All	46.0 (23.2–59.3)	52.5 (34.2–70.2)	35.7 (23.1–50.7)
	≥3	6.9 (2.4–18.3)	10.1 (3.3–26.7)	2.1 (0.3–13.7)
Nausea/vomiting	All	48.2 (32.5–64.2)	54.1 (38.7–68.7)	40
	≥3	5.8 (2.8–11.6)	6.3 (3.0–12.9)	0
Arrhythmia	All	25.7 (9.5–52.7)	37	15
	≥3	0	0	0
Fatigue	All	58.0 (39.3–74.7)	67.4 (56.7–76.5)	40
	≥3	2.6 (0.7–8.7)	2.7 (0.7–1.3)	0
Hemorrhage	All	NR	15.2 (9.0–24.5)	NR
	≥3	NR	1.8 (0.4–8.3)	NR
Hypertension	All	NR	2	NR
	≥3	NR	0	NR

*ERT* Endostatin combined with radiotherapy, *ECRT* Endostatin combined with concurrent chemoradiotherapy, *NR* Not reported

#### Radiation pneumonitis and esophagitis

The pooled frequencies of any grade and grade ≥ 3 radiation pneumonitis were 55.9 and 10.9% overall, 50.7 and 11.9% in the ECRT group, and 64.1 and 9.4% in the ERT group, respectively. The pooled frequencies of any grade and grade ≥ 3 radiation esophagitis were 77.4 and 11.6% overall, 89.7 and 12.2% in the ECRT group, and 55.5 and 9.4% in the ERT group, respectively.

#### Hematological toxicity

More than 10% of grade ≥ 3 hematological toxicities in all patients were neutropenia, leukopenia, and anemia, with incidences of 27.8, 35.5, and 10.5%, respectively. The pooled rates were 40.1% vs 2.1, 43.4% vs 0, and 12.3% vs 2.1%, respectively, in the ECRT and ERT groups. Rates of thrombocytopenia of grade ≥ 3 were 6.9, 10.1 and 2.1% for all patients, ECRT and ERT groups, respectively.

#### Other toxicities

Several other toxicities, including nausea, arrhythmia, fatigue, hemorrhage, and hypertension were additionally reported (Table 3). All of above AEs incidences of grade ≥ 3 were less than 10% for either all patients or for any of subgroups. Only one study reported AE of hypertension, in which patients received ECRT, with a frequency of 2% in any grade, and 0% in grade ≥ 3, respectively.

### Publication bias and sensitivity analysis

**Fig. 5:**
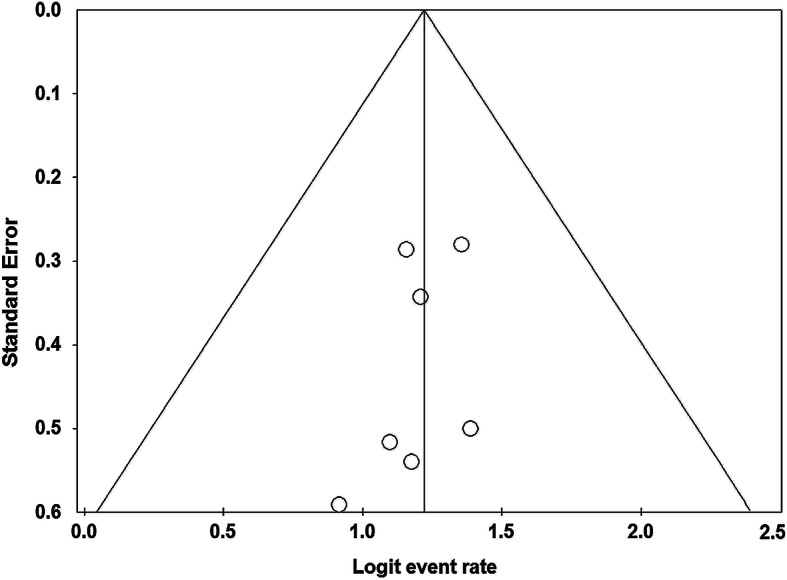
Funnel plot of publication bias for ORR. ORR: objective response rates

Publication bias was assessed for ORR according to Begg’s test and no significant publication bias was observed (Fig. 5). Besides, results of sensitivity analysis by omitting one study at a time did not substantially change the overall results.

## Discussion

CRT plus consolidation durvalumab is now considered standard of care for inoperable stage III NSCLC, but the optimal treatment strategies for the sequence and combination of CRT, immunotherapy, and even anti-angiogenic therapy are still being studied. Although data from prospective phase III randomized control studies evaluating the efficacy and safety of endostatin combined with RT or CCRT for patients with LA-NSCLC are lacking, our pooled analysis indicates that endostatin combined with CCRT or RT presents a promising treatment modality in treatment of LA-NSCLC; subgroups of ECRT and ERT have similar efficacy and survival benefit, but patients in the ERT subgroup had lower rates of toxicity.

Since tumor angiogenesis has been identified as a critical step in growth and metastasis of malignant solid tumors, anti-angiogenesis strategies have become established as an effective therapeutic approach [27–29]. Vascular endothelial growth factor (VEGF), a specific and potent angiogenic factor, contributes to the development of solid tumors by promoting angiogenesis. Several anti-VEGF or anti-VEGF-receptor (VEGFR) strategies have been developed to date, including neutralizing antibodies to VEGF/VEGFR, soluble VEGFR/VEGFR hybrids, and receptor tyrosine kinase inhibitors [30–32]. Chemotherapy combined with anti-angiogenic drugs [33–35], including bevacizumab (a VEGF-A monoclonal antibody), recombinant human endostatin, and ramucirumab (a VEGFR monoclonal antibody), has led to significantly prolonged survival, compared with chemotherapy alone, and is currently approved by the U.S. Food and Drug Administration (FDA) and/or China FDA for first- or second-line treatment of advanced NSCLC.

Solid tumors generally have characteristics of hypoxia and exhibit resistance to radiation to some extent, leading to failure of local control. Therefore, attempts to increase the sensitivity of RT via tumor oxygen enrichment present a novel direction for research [36, 37]. One of the most common factors causing hypoxia is inadequate vascular supply of the tumor, and thus sufficient blood vessel supply in the tumor microenvironment may be essential to improve the tumor radiation response for patients treated via RT [38]. Recombinant human endostatin is an endogenous broad-spectrum angiogenesis inhibitor produced by proteolytic cleavage of collagen XVIII that is suggested to interfere with the pro-angiogenic action of growth factors, such as basic fibroblast growth factor and VEGF. Preclinical studies have shown that recombinant human endostatin could transiently “normalize” the tumor vasculature to enhance efficiency of oxygen delivery and sensitivity to radiation treatment [39, 40]. Our pooled data indicate that combination of endostatin and RT with or without chemotherapy leads to better response rate, local control rate, and survival, demonstrating superior short- and long-term survival benefits, which are not inferior to the results of previous randomized controlled trials (RCTs) of CCRT (summarized in Table 4) [5, 41–44].

**Table 4:** The efficacy of concurrent chemoradiotherapy in previously reported phase II/III randomized controlled trials

Study	Number	CRT regimen	mPFS	PFS rate (%)		mOS	OS rate (%)		ORR	LCR (%)		
			(months)	1-yr	2-yr	(months)	1-yr	2-yr	(%)	1-yr	2-yr	Overall
RTOG 9410 [5]	195	RT + VP	NR	NR	NR	17	61.5	37.4	70.0	NR	NR	70
	187	RT + EP	NR	NR	NR	15.6	60.9	31.6	65.0	NR	NR	71
RTOG 0617 [41]	151	LDR + PC	11.8	49.2	29.1	28.7	80.0	57.6	NR	83.7	69.3	NR
	107	HDR + PC	9.8	41.2	21.4	20.3	69.8	44.6	NR	75.2	61.4	NR
	137	LDR + PC + Cet	10.8	44.3	24.2	25	76.2	56.3	NR	77.8	61.8	NR
	100	HDR + PC + Cet	10.7	46.3	27.5	24	71.1	50.1	NR	82.4	69.3	NR
PROCLAIM [42]	283	RT + PP	14.1	NR	NR	26.8	76.0	52.0	35.9	NR	NR	62.7
	272	RT + EP	9.8	NR	NR	25	77.0	52.0	33.0	NR	NR	54.2
CAMS [43]	95	RT + EP	14	56.8	29.5	23.3	74.1	48.4	73.7	NR	NR	NR
	96	RT + PC	12	50	17.7	20.7	80.2	43.8	64.6	NR	NR	NR
WJOG5008L [44]	54	RT + SP	14.8	55.6	29.6	40.9	87.0	75.6	76.9	NR	51	NR
	54	RT + VP	12.3	53.7	18.5	39	87.0	68.5	80.8	NR	28	NR

*CRT* Chemoradiotherapy, *RT* Radiotherapy, *LDR* Low dose radiation, *HDR* High dose radiation, *VP* Vinblastine plus cisplatin, *EP* Etoposide plus cisplatin, *PC* Paclitaxel plus carboplatin, *Cet* Cetuximab, *PP* Pemetrexed plus cisplatin, *SP* S1 plus cisplatin, *NR* Not reported, *PFS* Progression-free survival, *mPFS* Median progression-free survival, *OS* Overall survival, *mOS* Median overall survival, *ORR* Objective response rate, *LCR* Local control rate, *yr* Year

Although RTOG 0617 trial showed a superior median OS of 28.7 months, 69% patients in this study had stage IIIA disease [41]. In contrast, more than 50% patients in our pooled analysis had stage IIIB disease, which may be one of the factors contributing to survival differences. In a phase II trial involving 83% unresectable stage IIIB patients, endostatin combined with CCRT resulted in a median OS of 24 months [22]. In each of the RCTs listed in Table 4, over 50% of patients had a performance status (PS) score of 0; however, in our pooled analysis, only 28.5% of patients had a PS score of 0. In a phase II trial involving only 13.4% of patients with a PS score of 0, endostatin combined with CCRT resulted in median PFS and OS of 13.3 months and 34.7 months, respectively [18].

Recently, the PACIFIC study conducted in patients with unresectable stage III NSCLC showed a significant survival advantage with durvalumab consolidation therapy after CCRT [8], achieving a 3-year OS rate of 57% in the durvalumab group versus 43.5% in the control group. Based on this study, National Comprehensive Cancer Network guidelines have recommended this regimen as standard treatment for unresectable stage III NSCLC [45]. However, the optimal sequence and combination of CRT/RT and immunotherapy are being studied. Results from several phase II trials, such as the DETERRED and ETOP NICOLAS studies, have indicated that concurrent CRT with checkpoint inhibitors (ICIs) (atezolizumab/nivolumab) for the treatment of advanced NSCLC might be feasible and has no significant added toxicities over historical rates [46, 47]. Currently, many ongoing phase II/III clinical trials, such as PACIFIC2 (NCT03519971), KEYNOTE-799 (NCT03631784), EA5181 (NCT04092283), CheckMate73L (NCT04026412), etc., are evaluating the optimal treatment strategies of immunotherapy–radiotherapy combinations.

Although CCRT plays an indispensable role in the treatment of unresectable stage III NSCLC, some patients, especially the elderly or those with poor performance status who cannot tolerate toxicity induced by chemotherapy, have to receive sequential CRT or even RT alone [4, 5, 48]. Our pooled analysis indicated that patients treated with endostatin in combination with RT alone have comparable PFS (11.8 vs 11.2 months), OS (19.6 vs18.4 months), and ORR (76.1% vs 77.5%) to those administered endostatin with CCRT. In addition, pooled ORR data from the three prospective cohort studies showed that patients subjected to endostatin combined with RT had higher ORR (76.1% vs 61.7%), compared with the RT alone patient group. Therefore, combination therapy of RT and endostatin may be a promising strategy for LA-NSCLC patients with poor PS who cannot tolerate chemotherapy.

Of note, the duration and intervals of endostatin and radiotherapy combinations differed in clinical trials and may affect the outcomes (as shown in Table 1). Results from preclinical studies showed that endostatin treatment could transiently normalize the tumor vasculature by reducing microvessel density and increasing pericytic coverage of the vessel endothelium, thereby providing a time window (about 1 week) to enhance the sensitivity to RT; thus, RT delivery in this period resulted in maximal anti-tumor outcomes [15, 49]. CT perfusion imaging and hypoxia imaging suggested that the “time window” was within about 1 week after administration, during which endostatin improved blood perfusion and decreased hypoxia of lung cancer [14]. These studies provide an important experimental basis for combining endostatin with radiotherapy within the time window of 7 days (range, 5–10) after endostatin administration. In addition, given the short half-life of endostatin in vivo, CIV is considered a better delivery route to maintain a steady concentration and may improve its efficacy [49–51]. A recent study [52] which compared the outcomes of two phase II trials that involved different administration routes of endostatin combined with CCRT showed that endostatin at 7.5 mg/m^2^/24 h CIV for 5 days achieved higher 3- and 5-year OS rates (50.3, 41%) and safety than endostatin at 7.5 mg/m^2^/day IV for 7 days. Therefore, administration of 7.5 mg/m^2^/24h CIV for 5 days per 2 weeks, from 1 week pre-RT to the end of RT, could be a preferred scheme, on the basis of the current studies. However, the optimal duration and intervals of endostatin administration require further investigation.

In our pooled analysis, we observed that grade ≥ 3 AEs in the ECRT group were similar to those caused by CCRT reported previously (summarized in Table 5), indicating that addition of endostatin to CCRT did not obviously increase the main AEs. The pooled incidences of grade ≥ 3 radiation pneumonitis and radiation esophagitis were 10.9 and 11.6%, respectively, analogous to previous findings. Importantly, compared with the ECRT group, significantly lower rates of grade ≥ 3 AEs were observed in the ERT group, such as radiation pneumonitis (9.4% vs 11.9%), radiation esophagitis (9.4% vs 12.2%), nausea/vomiting (0% vs 6.3%), thrombocytopenia (2.1% vs 10.1%), neutropenia (2.1% vs 40.1%), anemia (2.1% vs 12.3%), and leukopenia (0% vs 43.4%).

**Table 5:** Adverse events of concurrent chemoradiotherapy in previously reported phase II/III randomized controlled trials

Study	CRT regimen	Leukopenia (%)		Neutropenia (%)		Thrombocytopenia (%)		Anemia (%)		Radiation pneumonitis (%)		Radiation esophagitis (%)	
		All	≥3	All	≥3	All	≥3	All	≥3	All	≥3	All	≥3
RTOG 9410 [5]	RT + VP	NR	83.9	NR	NR	NR	9.3	NR	11.8	NR	12.5	NR	22.2
	RT + EP	NR	68.4	NR	NR	NR	16.0	NR	18.8	NR	16.9	NR	44.9
RTOG 0617 [41]	LDR + PC	61.1	32.1	40.4	23.8	37.7	6.6	58.9	7.9	10.0	4.6	46.4	7.3
	HDR + PC	57.0	30.8	46.7	26.2	41.1	7.5	58.9	8.0	12.1	1.0	54.2	15.0
	LDR + PC + Cet	51.8	30.7	54.7	40.9	35.8	8.0	63.4	11.6	12.4	7.3	43.8	6.6
	HDR + PC + Cet	54.0	37.0	59.0	46.9	44.0	16.0	51.0	6.0	17.0	6.0	54.0	19.0
PROCLAIM [42]	RT + PP	36.7	22.6	42.8	24.4	55.0	40.3	40.3	8.8	17.0	1.8	48.1	15.5
	RT + EP	40.8	30.1	54.8	44.5	85.0	29.0	45.6	13.6	10.7	2.6	50.7	20.6
CAMS [43]	RT + EP RT + PC	95.8 92.7	30.5 20.7	NR NR	NR NR	12.7 5.2	0 0	24.2 13.5	0 0	76.8 72.9	7.4 8.3	87.0 84.0	20.0 6.3
WJOG5008L [44]	RT + SP	96.3	40.7	88.9	33.3	42.6	9.3	79.6	25.5	24.1	9.3	66.7	3.7
	RT + VP	100	79.6	94.4	75.9	22.0	3.7	88.9	27.8	20.4	7.4	74.1	0.0

*CRT* Chemoradiotherapy, *RT* Radiotherapy, *LDR* Low dose radiation, *HDR* High dose radiation, *VP* Vinblastine plus cisplatin, *EP* Etoposide plus cisplatin, *PC* Paclitaxel plus carboplatin, *Cet* Cetuximab, *PP* Pemetrexed plus cisplatin, *SP* S1 plus cisplatin, *NR* Not reported

Our pooled analysis has several limitations. Firstly, four in seven included studies belonged to single-arm trial and lacked a comparative control group, and another three of the studies were prospective cohort trials with a comparative control group, they were of non-random design and lacked sufficient data to facilitate effective analysis, Secondly, heterogeneity of the dose regimen or endostatin usage between studies was not taken into consideration, resulting in unstable merged findings. Thirdly, the current results suggest that endostatin combined with RT alone is comparable to endostatin with CCRT in terms of ORR, LCR, and survival. However, the differences in efficacy and safety between the two treatment methods remain to be established. Further well-designed prospective randomized controlled clinical trials are warranted to reach definitive conclusions.

Increasing interest has emerged in studying the feasibility of combined radiotherapy, antiangiogenic agents and ICIs. Current evidence suggests that antiangiogenic agents have the potential for increasing the response to immunotherapy by modulating the tumor microenvironment (TME) [53]. The IMpower150 study identified the synergic effect of antiangiogenic agents plus immunotherapy [54], in which patients in the atezolizumab plus bevacizumab and paclitaxel/carboplatin (ABCP) group achieved survival advantage over those in the bevacizumab plus paclitaxel/carboplatin (BCP) group. Similarly, preclinical study showed that endostatin plus anti-PD-1 also exerted a synergic effect on tumor growth in murine models of Lewis lung carcinoma by improving the TME and inducing autophagy [55]. An ongoing clinical trial (NCT04094909) is investigating the efficacy and safety of endostatin combined with chemotherapy and pembrolizumab as first-line therapy in patients with advanced or metastatic NSCLC. Despite the lack of clinical trials involving the combination therapy of endostatin, ICIs and RT/CRT, the synergic effect between endostatin and ICIs/RT will provide a potential way to improve clinical benefits for these patients when compared with current standard treatment.

## Conclusion

Based on this pooled data analysis, adding recombinant human endostatin to radiotherapy or concurrent chemoradiotherapy is an effective and less toxic method for the treatment of patients with unresectable LA-NSCLC. We suggest that concurrent administration of endostatin and CRT or RT presents a promising treatment approach for some patients in the era when CRT plus durvalumab has become the current standard of care. For patients who cannot tolerate CCRT and ICIs, endostatin combined with RT alone may be a good alternative, but for those patients who can tolerate CCRT but cannot tolerate ICIs, addition of endostatin to CCRT may become a more effective treatment strategy. High-quality prospective studies are needed to validate this suggestion. Given the synergistic antitumor effect of antiangiogenic agents and RT/ICIs on lung cancer, triple- or quadruple- combination therapy of endostatin, ICIs and RT/CRT for patients with inoperable stage III NSCLC might become a potential strategy in the future. However, multiple challenges regarding this combination remain to be addressed before it can be applied to clinical practice.

## Data Availability

The authors declare that all data generated or analyzed during this study are included in this article.

## Abbreviations

AEs: Adverse events
CCRT: Concurrent chemoradiotherapy
CI: Confidence interval
CIV: Continuous intravenous pumping
CRT: Chemoradiotherapy
ECRT: Endostatin plus concurrent chemoradiotherapy
ERT: Endostatin plus radiotherapy
FDA: Food and Drug Administration
ICIs: Immune checkpoint inhibitors
IV: Intravenous injection
LA-NSCLC: Locally advanced non-small cell lung cancer
LCR: Local control rate
NSCLC: Non-small cell lung cancer
ORR: Objective response rate
OS: Overall survival
PFS: Progression-free survival
PS: Performance status
RCTs: Randomized controlled trials
RT: Radiotherapy
TME: Tumor microenvironment
VEGF: Vascular endothelial growth factor
VEGFR: Vascular endothelial growth factor receptor
